# Aggressive natural killer cell leukemia or extranodal NK/T cell lymphoma? a case with nasal involvement

**DOI:** 10.1186/s13000-017-0636-1

**Published:** 2017-06-17

**Authors:** Xiaoke Jin, Youhai Xu, Jun Zhang, Guangxi Li, Dongping Huang, Yuqiong Yang, Hesheng He

**Affiliations:** 1 Laboratory of Hematopathology, Department of Hematology, the Affiliated Yijishan Hospital of Wannan Medical College, Wuhu, 241000 China; 2 Department of Hematology, the Affiliated Yijishan Hospital of Wannan Medical College, Zheshan West Road, Wuhu, 241000 China

**Keywords:** Aggressive natural killer cell leukemia, Large granular lymphocytic leukemia, Bone marrow involvement, NK cell neoplasms, NK/T cell lymphoma

## Abstract

**Background:**

Aggressive natural killer cell leukemia/lymphoma (ANKL) is a rare and highly aggressive NK cell neoplasm with a short clinical course and poor prognosis and is often misdiagnosed and confused with NK/T cell lymphoma (NKTL), which has a very different prognosis. Here, we present a case with nasal and bone marrow involvement, provide a literature review and make a differential diagnosis.

**Case presentation:**

A 41-year-old male presented nasal congestion pharyngalgia, palatal perforation, high fever and multiorgan dysfunction. Our diagnosis primarily relied on clinical features, the morphology and immunophenotype of the neoplastic cells and imaging studies. Characteristic large granular lymphocytes with azurophilic granules were visible in the bone marrow smears. In addition, the neoplastic cells expressed a typical immunophenotype, and the T cell receptor γ (TCR-γ) gene rearrangement analysis and presence of Epstein-Barr virus (EBV) were negative. The patient’s symptoms and signs were temporarily relieved after chemotherapy treatment, but after a short time, he underwent a rapid clinical decline and died 8 weeks later after admission due to multiorgan function failure.

**Conclusion:**

Our case demonstrates that to avoid a misdiagnosis, bone marrow analyses and other examinations should be performed early when a patient initially presents nasal lesions and other systemic symptoms. To the best of our knowledge, this may be the first reported case of ANKL with sternal tenderness.

## Background

Aggressive natural killer cell leukemia/lymphoma (ANKL) is a highly rare and extremely aggressive malignancy of NK cells initially classified as a type of large granular lymphocytic leukemia in 1986 [1]. It is prevalent in South America and Asia but represents less than 1% of all non-Hodgkin lymphoma (NHL) cases in North America and Europe. Men and women are equally affected, and the median age is 42 years old [2]. Because it is difficult to diagnose and treat ANKL during the early disease stages, ANKL is considered a catastrophic disorder due to the low morbidity rate, rapid clinical course and lack of unified diagnostic criteria. Here, we report a case of a middle-aged male who, at 41 years old, presented with initial symptoms of nasal congestion and pharyngalgia and was initially diagnosed with ANKL after examining the clinical features, the morphology and immunophenotype of neoplastic cells and available imaging. The symptoms were temporarily relieved after chemotherapy treatment, but a short time later, he experienced a rapid clinical decline and died of multiorgan function failure 8 weeks after admission.

## Case presentation

### Case report

**Fig. 1:**
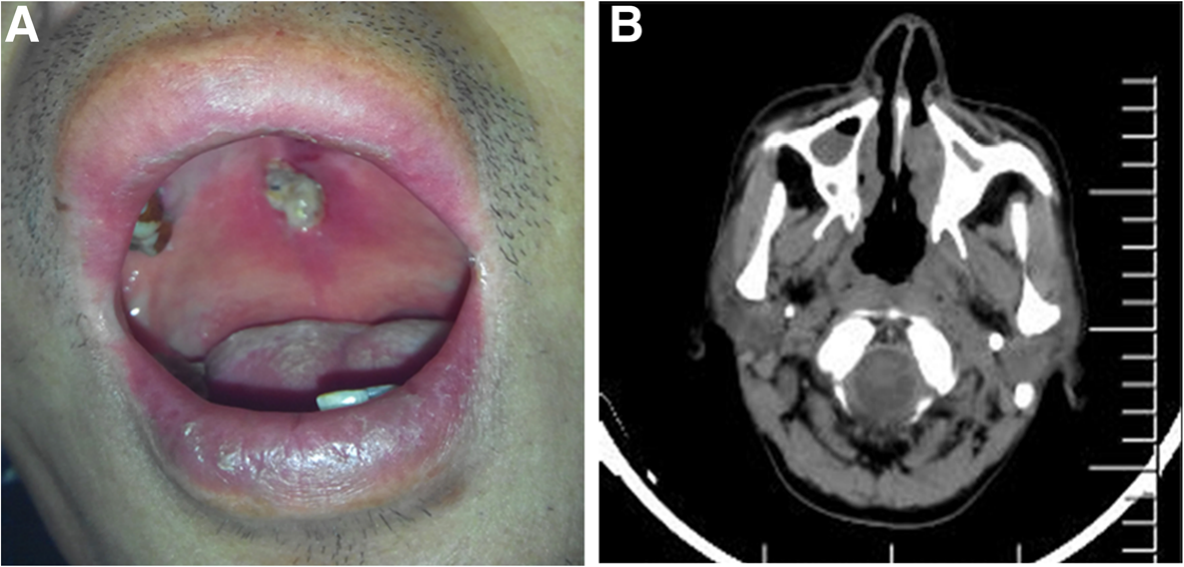
**a** An initial ulcer 1.5*0.5 cm in size with white purulent secretion and necrotic tissue on the surface could be seen on the palate. **b** Computed tomography scans revealed an occupied nasopharynx

**Fig. 2:**
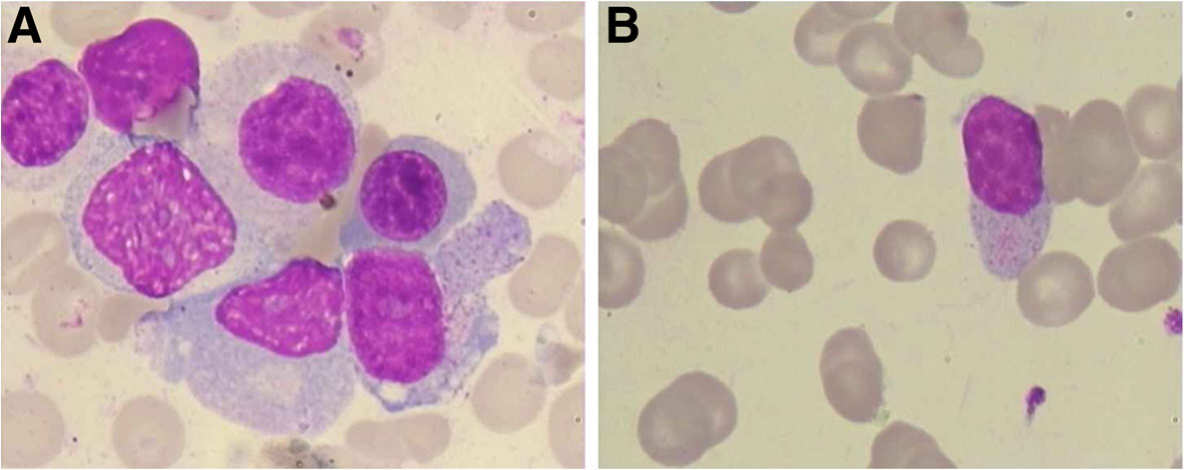
Bone marrow **a** and peripheral blood **b** smears after Wright-Giemsa staining. Abnormal lymphoid cells presented slightly basophilic cytoplasm, some which contained azurophilic granules, and slightly immature nuclei with either inconspicuous or distinct nucleoli

Our patient was a 41-year-old man who suffered from nasal congestion and pharyngalgia for a month before seeking treatment at the local hospital, but his symptoms were not resolved after receiving treatment. The patient was sent to our hospital because of anorexia, weakness, perspiration while sleeping and abdominal distension as well as nasal symptoms; after admission to our institution, he had a high fever of 40.1 °C, and the physical examination revealed several palpable superficial lymph nodes on his armpit and both sides of his groin. An initial ulcer 1.5 cm*0.5 cm in size with a white purulent secretion and necrotic tissue on the surface could be seen on the palate and ultimately perforated into the oral cavity 5 days later, after which the patient complained there was outflow from his nasal cavity when he drank liquids for a communication between the oral cavity and the nasal cavity was created (Fig. 1a). The abdomen was soft, and the liver was impalpable; however, the spleen was palpable 5 cm below the left costal margin. The blood parameters were as follows: (1) white blood cells (WBC), 2.5 × 10^9^/L; red blood cells (RBC), 3.91 × 10^12^/L; platelets (PLT) 93 × 10^9^/L, and hemoglobin (HGB) 108 g/L; (2) ALT, 57 U/L; AST, 70 U/L; lactate dehydrogenase (LDH), 642 IU/L; and C-reactive protein (CRP), 44.15 mg/L; and (3) activated partial thromboplastin time (APTT), 38.0 s. The ferroprotein levels were >1650 ng/ml (normal range 22–322 ng/ml), and the urine examination indicated proteinuria (+++); however, the patient was negative for Epstein-Barr virus (EBV). Computer tomography scans revealed an enlarged liver and spleen, an occupied nasopharynx and small nodules in the lung (Fig. 1b). B-mode ultrasonography showed enlarged lymph nodes on the neck, axilla and groin. The bone marrow smear revealed approximately 16% abnormal lymphoid cells that had a slightly basophilic cytoplasm; additionally, some of these cells presented azurophilic granules and slightly immature nuclei with inconspicuous or distinct nucleoli. The peripheral blood analysis indicated that 5% of the cells observed exhibited analogous traits as those in the abnormal lymphoid cells [Fig. 2(a, b)]. Flow cytometry of the bone marrow showed that the immunophenotype of the neoplastic cells was as follows: HLA-DR+, CD2+, cytoplasmic CD3+, CD7+, CD56+, surface CD3-, CD4-, CD8-, CD16-, CD57-, TCR α/β-, and TCR γ/δ-. In addition, the analysis of TCR-γ gene rearrangement in these cells was negative.

### Pathological findings

**Fig. 3:**
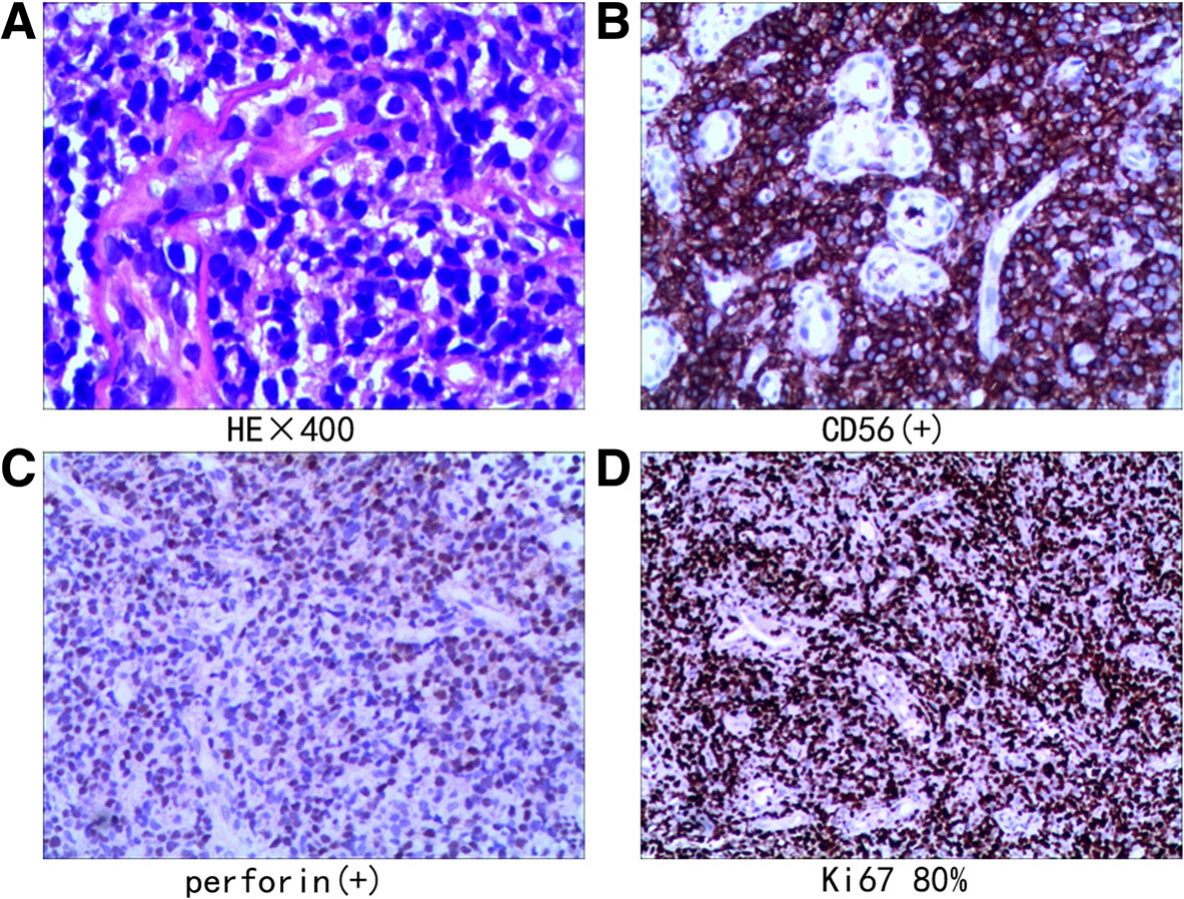
The pathological findings of the tissue. **a** HE staining, ×400. The tissue was identified as nasal mucosa and coagulative necrosis with infiltrating monomorphic medium-sized lymphoid cells. **b**, **c**, **d** Immunohistochemically, the neoplastic cells were CD56+ and perforin+, and is approximately 80% of the cells were Ki-67-positive

The patient’s nose was examined, and three small tissue samples were collected from nasal cavity for biopsy, two of which were microscopically assessed as nasal mucosa and coagulative necrosis whereas the third sample was gray-white in color, 0.5 cm × 0.2 cm × 0.2 cm in volume and contained infiltrated monomorphic medium-sized lymphoid cells. Immunohistochemically, these neoplastic cells were identified with the following markers: CD2+, cytoplasmic CD3+, CD20-, CD56+, EBER+, TIA-1+, perforin+, and Ki-67 > 80% [Fig. 3(a,b,c,d)]. Taken together with the results of the clinical and laboratory examinations, a final diagnosis of ANKL was made.

### Follow up

Aside from the nasal symptoms, the patient presented high fever, fatigue and weight loss when he was first admitted. He was treated with an EPOCH (etoposide, prednisone, vincristine, cyclophosphamide, and doxorubicin) plus thalidomide chemotherapy regimen starting on day 3 after the diagnosis was made. The fever, abdominal distension and poor appetite were temporary abated after treatment for a week, and the physical examination showed that the spleen was impalpable with simultaneous relief of the sternal tenderness. However, the symptoms in the nose and palate remained, and after a short time he underwent a rapid clinical decline with an enlarged spleen that was palpable 4 cm under the left costal margin. Unfortunately, he was died 8 weeks after initial admission due to multiorgan failure.

## Discussion

Here, we reported a patient who initially presented nasal congestion and pharyngalgia during the early stages of illness, and a diagnosis of ANKL was made after a series of examinations. It is worth noting that the clinical features and laboratory findings of the patient during the early phase showed many similarities with advanced stage extranodal NK/T cell lymphoma (ENKTL). Due to these similar characteristics, the boundary of the two diseases remains unclear, and it can be difficult to quickly and correctly diagnose these diseases.

ANKL is a rare and highly aggressive form of an NK cell neoplasm with an aggressive clinical course and poor prognosis. It was first reported as a type of large granular lymphocytic leukemia 28 years ago [1]. To date, fewer than 300 cases have been reported in the English literature worldwide [3–5]. According to the 2008 update of the World Health Organization (WHO) classification of tumors of hematopoietic and lymphoid tissues, another aggressive mature NK cell neoplasm is ENKTL, which is has a higher incidence than ANKL [6]. In addition, NK/T cell lymphoma can be further classified into the nasal and non-nasal subtype, which are clinically differentiated by the presence of absence of nasal involvement.

Relatively, ANKL is a more aggressive disease than ENKTL and often presents a more rapid clinical course despite the poor outcomes of both diseases [7]. As previously reported, most cases of ANKL were diagnosed from the presences of NK neoplastic cells in peripheral blood, bone marrow or tissue. However, a few ENKTL patients present with bone marrow involvement, but many ANKL patients can present with multiorgan involvement, including peripheral blood, bone marrow, live, and spleen, as well as destruction of the facial midline. In addition, the number of leukemia cells in the bone marrow and peripheral blood may fluctuate over a comparatively large range [8] which may result in a misdiagnosis. In addition, when the nose and nasopharynx are initially involved, it is much harder to confirm the type of disease, which may indicate the prognosis of the patient.

Our case had the following features. First, our patient was a 41-year-old man who suffered from nasal congestion, pharyngalgia, high fever and weight loss. Further examinations revealed widespread systemic dissemination of disease that involved clinical features of several organs such as the enlargement of liver, spleen and lymphonodus. Additionally, peripheral blood and bone marrow smears were concurrently analyzed under a microscope. ENKTL (especially the nasal subtype) is a localized disease with and older median age and often involved the nose and nasopharynx; however, the most common sites of ANKL infiltration include the skin, gastrointestinal tract, salivary glands, testis and other organs. Cases could be diagnosed as a non-nasal type before the lesion could be observed by positron emission tomography computed tomography (PET/CT) [9], implying that the cases we diagnosed as non-nasal type lymphoma prior to the advent of modern imaging technology may be either nasal type lymphoma or ANKL. ENKTL with bone marrow involvement is rare, bone marrow as involved in fewer than 10% patients initially diagnosed with ENKTL presented bone marrow involvement, and only 6 cases have been reported to date [10–12]. Cases have been reported with hepatosplenomegaly, splenic rupture and bone marrow fibrosis [13–15], but there are no reports describing the status of sternum to date; however, our patient complained of obvious bone pain in bone, which is an important physical sign that is often ignored. Therefore, this may be the first reported case with sternal tenderness to the best of our knowledge.

Second, the neoplastic cells observed in the blood and bone marrow were initially thought to be large granular lymphocytes, which conformed to the features of NK cells. As these cells reported a loss of CD16 expression [4] during the immunophenotype analysis, the lack of TCRα/β and TCRγ/δ helped to exclude T cell leukemia/lymphoma, and the positive Ki-67 rate greater than 80% indicated the malignant status. According to studies on ANKL and ENKTL, the immunophenotypic characteristics of the two diseases appear to be similar. Suzuki R et al. retrospectively analyzed the immunophenotype of 22 patients with ANKL and 150 patients with ENKTL and demonstrated that the expression of cytoplasmic CD3 was significantly higher in patients ENKTL (82% versus 43%, *P* = 0.009) but that of CD16 was lower (22% versus 75%, *P* < 0.001) in ANKL, which may help differentiate these diseases on some level [5]. In addition, the patient in our case report was negative for EBV, which is rare but exists—7 cases were recently reported by Nicolae A et al. [16], who indicated that EBV-negative cases tend to occur in older patients and were clinically and pathologically indistinguishable from EBV-positive ANKL with a similar fulminant clinical course.

Third, the patient suffered many pathogenic manifestations aside from his nasal symptoms, including pancytopenia hepatosplenomegaly, lymphadenopathy and sternal tenderness, all of which were observed when he first admitted. The bone marrow and peripheral blood analyses were verified using morphological and flow cytometry examinations, and the results were consistent with the features of stage IV (Ann Arbor staging system) ANKL. Different from ENKTL, most ANKL patients are diagnosed with stage III/IV disease [2], and no standard chemotherapy is currently available. Although ANKL was initially thought to be potentially chemosensitive disease, the treatment with high-dose chemotherapy and HSCT (hematopoietic stem cell transplantation) are still recommended. Our patient was treated with EPOCH plus thalidomide, and his symptoms were briefly ameliorated, but after a short time, he underwent a rapid clinical decline and no longer qualified for HSCT. During his treatment, the disease exhibited chemosensitivity as previously reported [17].

The case demonstrated that without systemic examinations and analysis, the primary sites of presentation could lead to wrong diagnosis and lose the chance for appropriate treatment. When combining the results of the morphology, immunophenotype, pathology, and immunohistochemical analysis with the clinical presentation, a final diagnosis of ANKL was indicated.

## Conclusion

We report a case of ANKL with nasal involvement and multiorgan dysfunction upon initial diagnosis which may be the reported first case of ANKL with sternal tenderness—an important physical sign that is often ignored. ANKL can be misdiagnosed and confused with ENKTL, which has a different prognosis. A series of physical, morphological, and pathological examinations as well as modern imaging technology (PET/CT et al.) may help to correctly diagnose treat patients with an effective chemotherapy with the goal of providing a chance to undergo HSCT as soon as possible.

## Abbreviations

ANKL: Aggressive natural killer cell leukemia/lymphoma
APTT: Activated partial thromboplastin time
CRP: C-reactive protein
EBV: Epstein-Barr virus
ENKTL: Extranodal NK/T cell lymphoma
EPOCH: Etoposide, prednisone, vincristine, cyclophosphamide, and doxorubicin
HGB: Hemoglobin
HSCT: Hematopoietic stem cell transplantation
LDH: Lactate dehydrogenase
NHL: Non-Hodgkin lymphoma
PET/CT: Positron emission tomography computed tomography
PLT: Platelets
RBC: Red blood cells
TCR-γ: T cell receptor γ
WBC: White blood cells
WHO: World Health Organization
